# Vein of Marshall ethanol infusion improves ablation outcomes in patients with persistent atrial fibrillation

**DOI:** 10.1016/j.hroo.2025.01.020

**Published:** 2025-02-10

**Authors:** Mohammed Al-Sadawi, Rushil N. Shah, Amrish Deshmukh, Jackson J. Liang, Krit Jongnarangsin, Fred Morady, Hakan Oral, Aman Chugh, Michael Ghannam

**Affiliations:** Division of Cardiovascular Medicine, Department of Electrophysiology, University of Michigan, Ann Arbor, Michigan

**Keywords:** Vein of Marshall, Ethanol ablation, Catheter ablation, Nonparoxysmal atrial fibrillation, Posterior wall ablation

## Abstract

**Background:**

Vein of Marshall ethanol infusion (VoMEI) may improve outcomes among patients with persistent atrial fibrillation (AF) undergoing catheter ablation procedures. Prior investigations used heterogeneous ablation strategies, limiting the understanding of VoMEI utility.

**Objective:**

The study sought to examine the safety and efficacy of a uniform ablation approach utilizing VoMEI compared with patients undergoing pulmonary vein isolation (PVI) only or PVI and posterior wall isolation (PWI).

**Methods:**

Patients undergoing first-time ablation for persistent AF utilizing VoMEI with PVI, PWI, and ablation of induced macro–re-entry arrhythmias were included. Two propensity-matched control groups (PVI only and PVI + PWI) were identified with 2:1 matching. Safety and efficacy rates between the groups were examined.

**Results:**

A total of 155 patients were included (VoMEI, n = 31; PVI, n = 62; PVI + PWI, n = 62), consisting of 98 (63%) males 66 ± 10 years of age, with left atrial diameter 49 ± 7 mm, ejection fraction 50 ± 16%, and follow-up time 3.2 ± 2.2 years, with no demographic differences between the groups (*P >* .05). The 1-year rate of freedom from atrial arrhythmias after a single procedure was 84%, 67%, and 54% for patients undergoing VoMEI, PVI only, and, PVI + PWI, respectively (log rank *P* = .021). Patients who underwent VoMEI had improved outcomes compared with patients who did not (hazard ratio 0.32, 95% confidence interval 0.12–0.78, *P =* .01), with fewer repeat procedures (10% vs 37%, *P <* .01). Procedure, radiofrequency, and fluoroscopy times were greater in the VoMEI groups (*P >* .05).

**Conclusion:**

Among patients with persistent AF, an ablation strategy incorporating VoMEI improved long-term ablation outcomes compared with groups of propensity-matched patients undergoing PVI only or PVI + PWI.

**Key Findings**

▪ Vein of Marshal ethanol infusion (VoMEI) may improve outcomes among patients undergoing ablation of persistent atrial fibrillation. The optimal ablation strategy which incorporates VoMEI remains unclear.
▪ In this propensity-matched study, a standardized approach utilizing VoMEI with pulmonary vein isolation (PVI), posterior wall isolation (PWI), mitral isthmus block, and ablation of induced macro–re-entrant arrhythmias was shown to be superior to PVI only or PVI + PWI ablation.
▪ Strategies utilizing VoMEI incur added procedural complexity over PVI only or PVI + PWI ablation; however, there were no differences in adverse procedural outcomes.

## Introduction

Long-term outcomes in patients undergoing catheter ablation of persistent atrial fibrillation (AF) remain suboptimal, with many ablation strategies failing to demonstrate benefit beyond pulmonary vein isolation (PVI) alone.^1^ In a randomized controlled trial, a strategy incorporating vein of Marshall ethanol infusion (VoMEI) was shown to improve outcomes as compared with conventional radiofrequency (RF) ablation, which included PVI and ablation of other targets.^2^ Patients in both arms underwent mapping and ablation of multiple other targets including the superior vena cava, right atrium, and electrogram-guided ablation. The incremental effect of incorporating these sites is unknown. The purpose of this study is to compare outcomes utilizing a more standardized approach incorporating VoMEI to patients undergoing either PVI only or PVI with posterior wall isolation (PWI) among patients undergoing first-time ablation of persistent AF.

## Methods

### Study population

**Figure 1:**
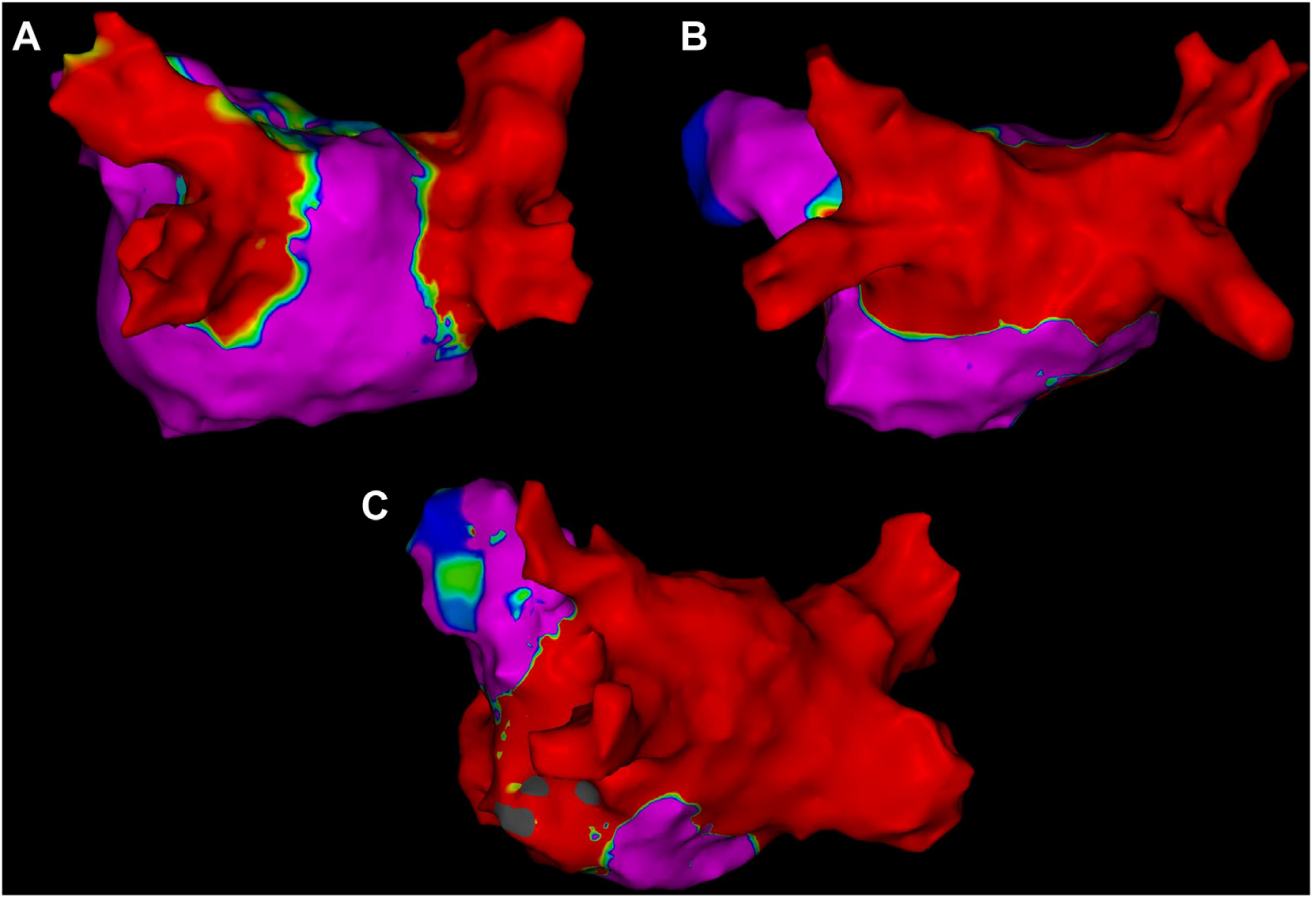
Ablation approaches for persistent atrial fibrillation. Postablation 3-dimensional electroanatomic maps displayed in the posteroanterior projections. Ablation strategies among the 3 groups including patients who underwent pulmonary vein isolation only (A), patients with pulmonary vein isolation and posterior wall isolation (B), or patients with pulmonary vein isolation, posterior wall isolation, and vein of Marshall ethanol infusion along with additional ablation to ensure mitral isthmus block.

This was a retrospective, single-center study of consecutive patients undergoing first-time RF ablation and VoMEI for persistent AF from January 1, 2022, to June 1, 2023. Propensity matching in a 2:1 fashion was used to identify patients undergoing (1) PVI only or (2) PVI + PWI (Figure 1), and patients were drawn from an historic cohort undergoing ablations beginning in 2017. The total group consisted of 155 patients (66 ± 10 years, 98 [63%] male, ejection fraction 50 ± 15%, left atrial [LA] diameter 49 ± 7 mm, CHA_2_DS_2_-VASc [congestive heart failure, hypertension, age ≥75 years, diabetes mellitus, prior stroke or transient ischemic attack or thromboembolism, vascular disease, age 65–74 years, sex category] 2.9+1.5) including 61(39%) who had previously failed antiarrhythmic medications. Ten patients had a history of long-standing persistent AF (PVI only, n = 4 [6.5%]; PVI + PWI, n = 3 [4.8%]; VoMEI, n = 3 [9.7%]; *P =* .7). The study protocol was approved by the institutional review board of the University of Michigan, and the research reported in this paper adhered to the Helsinki Declaration.

### Electrophysiology study and ablation

All patients provided informed written consent. All antiarrhythmic drugs were discontinued 4 to 5 half-lives before the study. Amiodarone was discontinued 2 months prior to the procedure. Oral anticoagulation with warfarin was not interrupted. Patients taking a direct oral anticoagulant were asked to withhold the medication on the day of the procedure. The ablation procedure was performed under general anesthesia. Vascular access was obtained through the femoral veins. A multipolar catheter was positioned in the coronary sinus (CS) and was used for recording and atrial pacing. Systemic anticoagulation was achieved with intravenous heparin to maintain an activated clotting time of >350 seconds. A 3-dimensional mapping system (CARTO; Biosense Webster) was used to guide catheter navigation and ablation. An open-irrigation, 3.5-mm-tip deflectable catheter was used for mapping and ablation. Bipolar electrograms were recorded at a band pass of 30 to 500 Hz (EPMedSystems). The esophagus was delineated with a radio-opaque marker. RF energy was applied at a maximum power output of 25 to 50 W at a flow rate of 30 mL/min and a maximum temperature of 45 °C. When ablation was performed near the pulmonary vein ostia, or in the posterior LA, the power was reduced to 25 W at a flow rate of 17 mL/min. Power was limited to 20 W during energy application in the CS. A minimum of 10 g contact force and a 10 Ω impedance drop was targeted during ablation.

**Figure 2:**
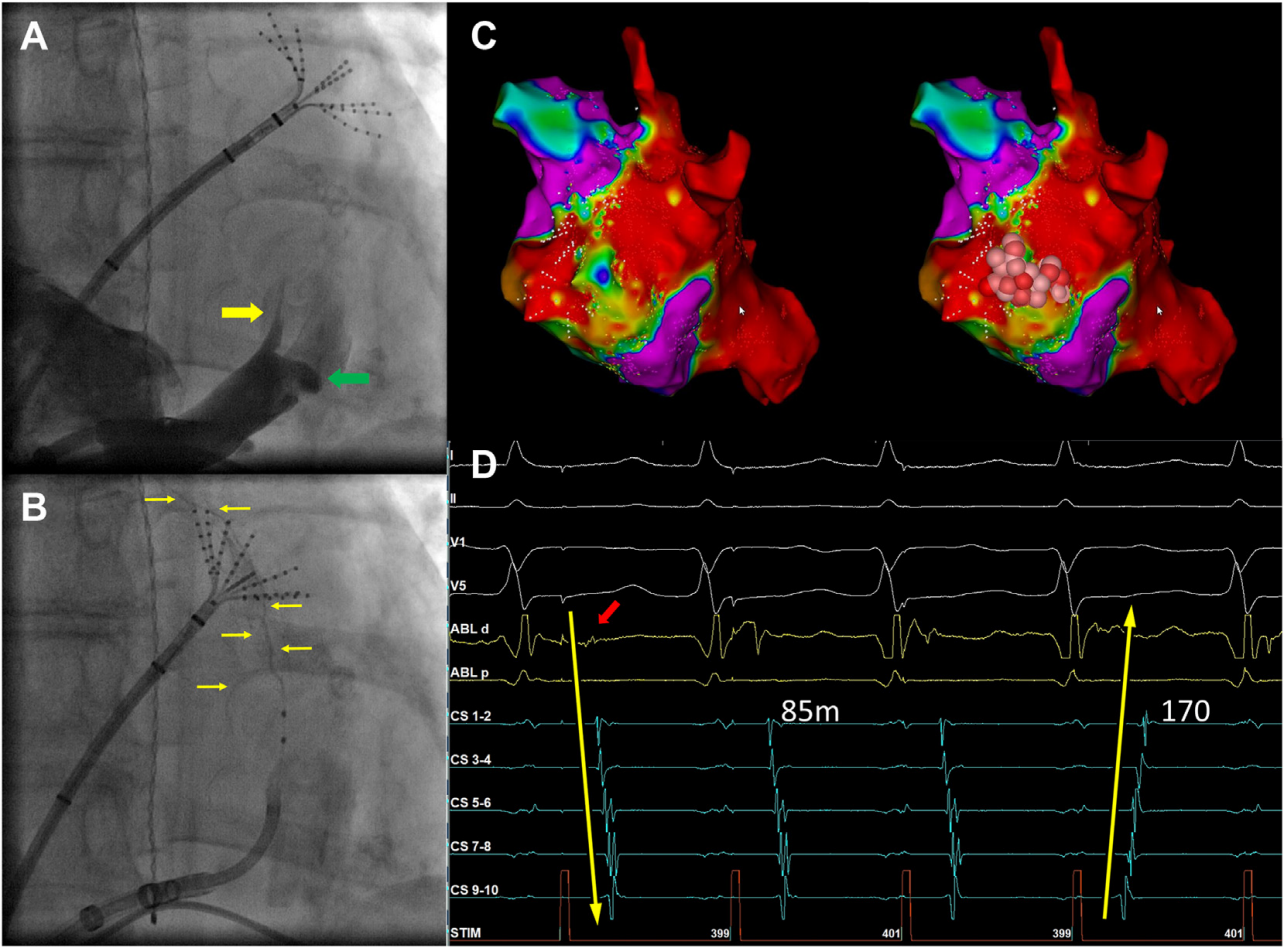
Vein of Marshall ethanol infusion. Procedural steps of vein of Marshall ethanol infusion is demonstrated in a patient undergoing first-time ablation of persistent atrial fibrillation. A: Fluoroscopy in the anterior posterior projection is shown, and venography of the coronary venous system demonstrates the vein of Marshall (yellow arrow) branching posteriorly and superiorly from the coronary venous system, proximal to the valve of Vieussens (green arrow). B: The vein of Marshall is instrumented with a guiding catheter, angioplasty wire, and occlusive balloon. Selective venography shows the course of the vein of Marshall (yellow arrows), and a mapping catheter is placed in the left atrial appendage to serve as an anatomic landmark. Ethanol is slowly injected in the distal and proximal sections of the vessel. C: An electroanatomic map of the lateral mitral isthmus after ethanol ablation shown, showing a small area of preserved voltage near the mitral annulus. Endocardial ablation lesions were applied in this area (red tags). D: A coronary sinus catheter was placed proximal to the endocardial lesions and pacing was performed from the left atrial appendage during endocardial ablation. A small endocardial signal is shown (red arrow), preceding the earliest coronary sinus signals. During ablation, this signal disappears resulting in reversal of the coronary sinus activation pattern, with an increase in the left atrial appendage to distal coronary sinus activation time from 85 ms to 170 ms, consistent with mitral isthmus block.

Patients in the study group underwent PVI followed by PWI via the creation of a roof line, inferior line, and ablation of residual potentials within the posterior wall. Bidirectional block was confirmed with mapping and pacing maneuvers. VoMEI was then performed using previously published methods.^2^^,^^3^ Briefly, the CS was instrumented either from the internal jugular or femoral vein, and the vein of Marshall (VoM) was demonstrated on angiography. The VoM was cannulated with an angioplasty wire (PROWATER; Asahi Intecc) supported by an over-the-wire balloon (1.5–2.5 mm diameter). The balloon was inflated in to ensure complete occlusion of the VoM. Ethanol (up to 2–3 cm^3^ per segment; maximum total of 10 cm^3^) was infused slowly (1 cm^3^ per minute) into the distal and proximal portions of the VoM. Upon completion, additional endocardial and epicardial (CS) ablation was performed as needed to attain mitral isthmus block assessed by standard criteria (Figure 2). Cavotricuspid isthmus (CTI) ablation was performed in patients with a clinical history of typical atrial flutter, or with those who had typical atrial flutter induced with burst atrial pacing. Additional induced or spontaneous macro–re-entry arrhythmias were ablated. The matched control groups consisted of patients who underwent PVI only or PVI + PWI.

### Postablation management and follow-up

Patients were monitored on a telemetry unit overnight. Patients were seen in an outpatient clinic 3 months after the ablation procedure and every 3 to 6 months thereafter. Patients on long-term anticoagulation were enrolled in a pharmacist-led anticoagulation clinic for monitoring. Rhythm status was assessed via a 30-day event monitor 12 months after the ablation procedure. Recurrence was defined as sustained (>30 seconds) symptomatic or asymptomatic AF/atrial tachycardia (AT) after the 3-month blinding period.

### Statistical analysis

Continuous variables were expressed as mean ± SD and categorical variables were expressed as counts and percentages. Data were compared using Fisher’s exact test or chi-square test for categorical variables and the Student’s *t* test or Mann-Whitney test for continuous variables as appropriate. The 2 control groups were identified with 2:1 propensity matching using the nearest-neighbor matching method in the MatchIt packing within R version 4.4.1 (R Foundation for Statistical Computing). Variables selected for matching include age, sex, body mass index, ejection fraction, and LA diameter. Survival analysis was performed to examine long-term AF/AT recurrence among the 3 groups, and the main outcome was 1-year arrhythmia-free survival after a single ablation procedure. A 2-tailed *P <* .05 indicated statistical significance. All statistical analyses were performed using R.

## Results

**Figure 3:**
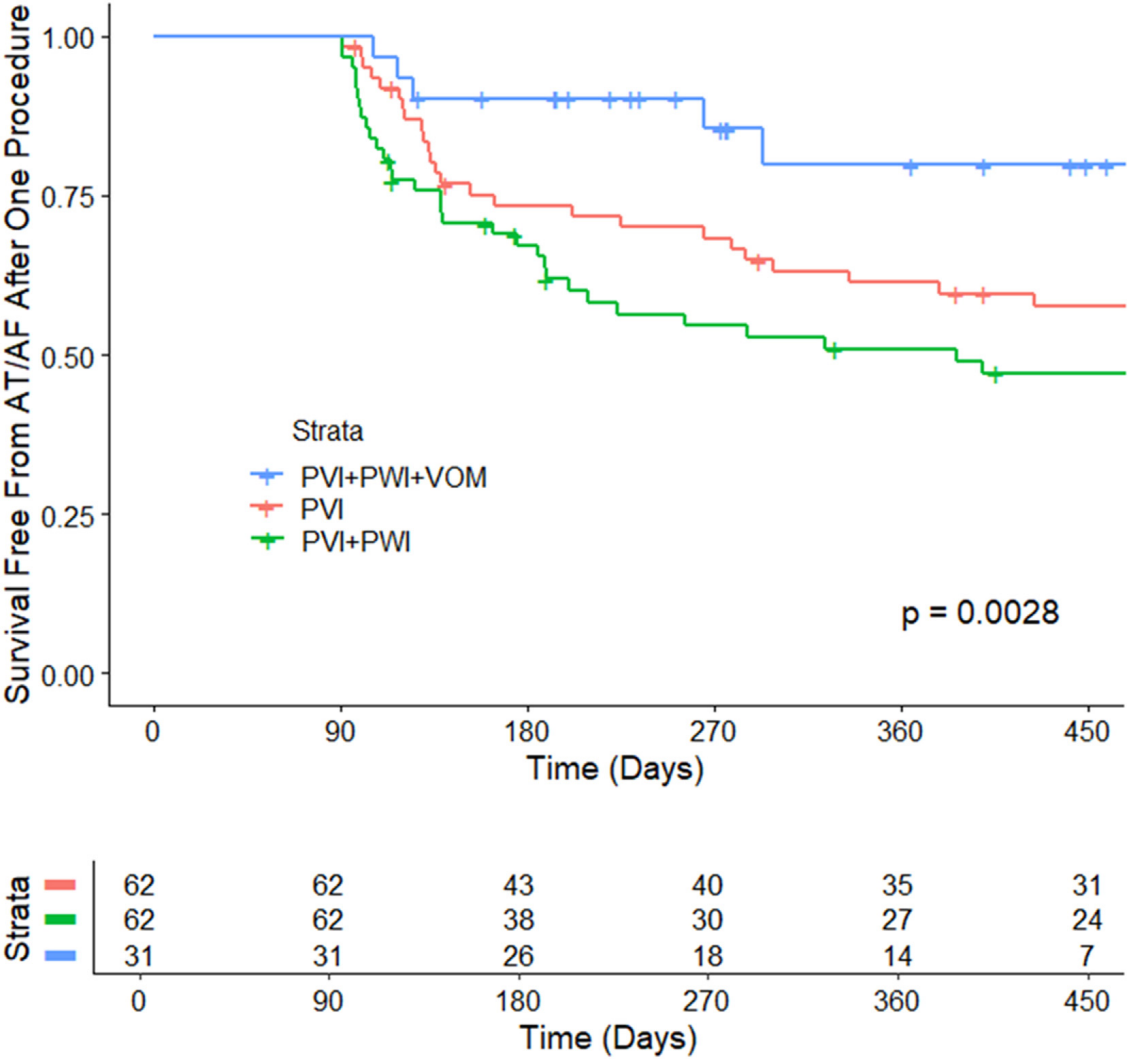
Long-term arrhythmia-free survival after a single ablation procedure among patients with nonparoxysmal atrial fibrillation (AF). AT = atrial tachycardia; PVI = pulmonary vein isolation; PWI = posterior wall isolation; VoM = vein of Marshall.

There were no differences in the baseline characteristics among patients who underwent PVI only (n = 62), PVI + PWI (n = 62), or a strategy incorporating VoMEI (n = 31) (*P* > .05 for all) (Table 1). The endpoints of PVI with or without PWI were achieved in all patients in the latter groups. After confirming isolation of the PVs and posterior left atrium (LA) in the VoMEI group, mitral isthmus conduction block was attained in 29 (94%) of 31 patients (Figure 3). All required endocardial ablation at the lateral mitral annular region, and 25 required further RF ablation in the CS. Fourteen patients in the VoMEI group underwent CTI ablation for inducible typical atrial flutter. Patients in the VoMEI group underwent anterior wall ablation (n = 2) or LA appendage ablation without isolation (n = 3) for easily inducible ATs encountered during the ablation procedure. There were no major procedural complications such as stroke, hemorrhage, perforation, or phrenic nerve injury. LA appendage isolation did not occur in any of the patients. The procedural times, radiofrequency times, and fluoroscopic times are shown in Table 2.

**Table 1:** Baseline patient characteristics

	Total (n = 155)	PVI only (n = 62)	PVI + PWI (n = 62)	PVI + PWI + VoM (n = 31)	*P*
Age, y	65.95 ± 10.11	65.80 ± 9.49	65.08 ± 11.11	67.98 ± 9.20	.426
Male	98 (63.2)	40 (64.5)	39 (62.9)	19 (61.3)	.953
BMI, kg/m^2^	34.58 ± 6.52	34.93 ± 6.69	34.50 ± 6.46	34.06 ± 6.48	.824
LA diameter, mm	48.50 ± 7.40	48.77 ± 6.95	48.23 ± 8.02	48.52 ± 7.22	.919
EF, %	49.74 ± 15.50	50.00 ± 15.21	49.58 ± 17.38	49.52 ± 12.17	.985
HTN	118 (76.1)	47 (75.8)	51 (82.3)	20 (64.5)	.166
DM	40 (25.8)	20 (32.3)	16 (25.8)	4 (12.9)	.132
CVA	13 (8.4)	5 (8.1)	6 (9.7)	2 (6.5)	.863
CAD	35 (22.6)	14 (22.6)	14 (22.6)	7 (22.6)	1
CABG	7 (7.5)	0 (0)	5 (8.1)	2 (6.5)	1
HCM	4 (2.6)	1 (1.6)	3 (4.8)	0 (0.0)	.322
CHF	51 (32.9)	22 (35.5)	18 (29.0)	11 (35.5)	.704
CHA_2_DS_2_-VASc	2.9 ± 1.5	3.00 ± 1.54	2.94 ± 1.37	2.73 ± 1.51	.712
Prior AAD	61 (39.4)	19 (30.6)	26 (41.9)	16 (51.6)	.129
BB	120 (77.4)	47 (75.8)	46 (74.2)	27 (87.1)	.346
CCB	46 (29.9)	18 (29.0)	24 (38.7)	4 (13.3)	.044
ACE inhibitor	62 (40.0)	29 (46.8)	23 (37.1)	10 (32.3)	.337
ARB	39 (25.2)	14 (22.6)	15 (24.2)	10 (32.3)	.583
Digoxin	12 (7.7)	4 (6.5)	7 (11.3)	1 (3.2)	.346
Aspirin	21 (13.5)	4 (6.5)	11 (17.7)	6 (19.4)	.106
Statin	87 (56.1)	32 (51.6)	35 (56.5)	20 (64.5)	.496
Warfarin	20 (12.9)	9 (14.5)	11 (17.7)	0 (0.0)	.049

Values are mean ± SD or n (%).

AAD = antiarrhythmic drug; ACE = angiotensin-converting enzyme; ARB = angiotensin receptor blocker; BB = beta-blocker; BMI = body mass index; CAD = coronary artery disease; CABG = coronary artery bypass grafting; CCB = calcium channel blocker; CHA_2_DS_2_-VASc = congestive heart failure, hypertension, age ≥75 years, diabetes mellitus, prior stroke or transient ischemic attack or thromboembolism, vascular disease, age 65–74 years, sex category; CHF = congestive heart failure; CVA = cerebrovascular accident; DM = diabetes mellitus; EF = ejection fraction, HTN = hypertension; HCM = hypertrophic cardiomyopathy, LA = left atrium; PVI = pulmonary vein isolation; PWI = posterior wall isolation; VoM = vein of Marshall.

**Table 2:** Procedural characteristics

	Total (n = 155)	PVI only (n = 62)	PVI + PWI (n = 62)	PVI + PWI + VoM (n = 32)	*P*
Procedure time, min	211.80 ± 73.60	182.27 ± 66.11	217.85 ± 72.87	241.61 ± 72.25	.001
RF time, min	47.96 ± 20.20	37.52 ± 17.45	50.14 ± 17.01	58.42 ± 23.27	<.001
Fluoroscopy time, min	23.91 ± 14.19	24.82 ± 17.63	18.31 ± 7.90	33.84 ± 13.13	<.001

PVI = pulmonary vein isolation; PWI = posterior wall isolation; RF = radiofrequency; VoM = vein of Marshall.

### Follow-up

Patients were followed for a median of 3.2 ± 2.2 years, during which 84 (55%) of 155 patients had recurrence of AF/AT. There were 11 deaths over the follow-up period, due to heart failure (n = 5), cancer (n = 3), sudden cardiac death (n = 2), muscular dystrophy (n = 1), and stroke (n = 1). At the end of the follow-up period, 37 (24%) of 155 patients remained on antiarrhythmic drugs (amiodarone, n = 14; dofetilide, n = 6; dronedarone, n = 3; flecainide, n = 5; propafenone, n = 2; sotalol, n = 7). Specifically, antiarrhythmic medications were prescribed in 5 (16%) of 31, 21 (34%) of 62, and 11 (18%) of 62 patients in the VoMEI, PVI only, and PVI + PWI groups respectively, with no differences among the 3 groups (*P =* .05).

The long-term single procedure rate of arrhythmia-free survival was 67% for patients undergoing PVI only, 54% for patients undergoing PVI + PWI, and 84% for patients undergoing VoMEI (log-rank *P* = .021) (Figure 3). Long-term freedom for arrhythmias or antiarrhythmic drug use was 32% for PVI only, 27% for PVI + PWI, and 74% for VoMEI (log-rank *P <* .003). There were no differences in arrhythmia-free survival among patients who underwent PVI only compared with PVI + PWI (hazard ratio [HR] 1.49, 95% confidence interval [CI] 0.913–2.45, *P =* .11). Patients who underwent an ablation strategy incorporating VoMEI had improved outcomes compared with patients who did not (HR 0.32, 95% CI 0.12–0.78, *P =* .01).

Repeat procedures were performed in 49 (32%) patients, including in 18 (29%) patients in the PVI only group, 28 (45%) patients in the PVI + PWI group, and 3 (10%) patients in the VoMEI group. Patients who underwent a VoMEI approach were less likely to require a repeat procedure as compared with the others (10% vs 37%, *P <* .01). One of these patients underwent ablation for typical atrial flutter. One patient had recurrence of AF and underwent reisolation of posterior wall along with ablation at the base of LA and right atrial appendages. One patient had a Bachmann’s bundle–related AT, which terminated with the creation of anterior line. Mitral isthmus block could not be achieved during the initial procedure in this patient but was attained with further endocardial/CS ablation at the redo session. The remaining 2 patients who had attained mitral block at the first procedure were found to have durable block during the redo session.

Eighteen patients with PVI only underwent repeat ablation for AF (n = 14) or AT (n = 4). Clinical ATs in this group included roof-dependent flutter (n = 2), CTI flutter (n = 1), and perimitral flutter (n = 1). Fourteen (77%) of 18 patients required reisolation of the PVs. One patient underwent repeat PVI only, the remaining 17 underwent additional PWI, CTI, or linear LA ablation.

Twenty-eight patients with PVI + PWI underwent repeat ablation for AF (n = 17) or AT (n = 11). The mechanism of organized arrhythmias included mitral isthmus–dependent AT (n = 6), roof-dependent AT (n = 4), and LA septal flutter AT (n = 1). Twenty-five (90%) of 28 patients required reisolation of either the pulmonary vein or posterior wall. Eight patients underwent only reisolation of the pulmonary veins and/or posterior walls, with the remaining undergoing additional linear LA ablation. No patient had electrical isolation of the LA appendage. After allowing for multiple procedures, the 1-year rate of arrhythmia-free survival was 84%, with no differences between the groups (log rank *P >* .05).

## Discussion

Among patients with persistent AF, an ablation strategy incorporating VoMEI was associated with superior long-term outcomes after a single procedure, as compared with patients undergoing PVI only or PVI + PWI. The addition of chemical ablation to conventional targets such as the pulmonary veins and posterior LA was not associated with a higher risk of adverse outcomes, or other concerns such as LA appendage isolation; however, procedural, RF, and fluoroscopic times were greater in the VoMEI groups. The suboptimal outcomes in the non-VoMEI groups are consistent with results from other studies showing modest returns with more limited ablation strategies, targeting only the pulmonary veins, even with adjunctive posterior LA ablation.^4^^,^^5^ Although arrhythmia-free outcomes after multiple procedures were similar among the 3 groups, patients in the VoMEI group were less likely to require repeat ablations.

### Mechanisms of VoMEI

The mitral isthmus has been long recognized as an important ablation target in patients with persistent AF.^6^^,^^7^ The progressive prolongation of AF cycle length and termination of AF during linear ablation at the mitral isthmus supports the fibrillatory potential of the lateral LA.^8^ Although linear ablation at the isthmus helps eliminate AF, it often is met with re-entrant AT, most often due to perimitral re-entry. It should be noted that mitral isthmus–dependent atrial flutter may be encountered even in patients who do not undergo empiric ablation of the LA isthmus. Although conduction block can be achieved in the majority of patients after additional ablation in the CS, recovery is not uncommon, resulting in recurrence and repeat ablation procedures. The salutary effective of chemical ablation of the VoM seems to be in large part due to its contribution to durable conduction block across the mitral isthmus. Additionally, an increased area of ablated tissue and durable segmentation of the atria through VoMEI may result in atrial “debulking,” interrupting fibrillatory wavefronts, which may contribute to the perpetuation of persistent AF.^9^

Through elimination of LA ridge tissue and epicardial connections, VoMEI has been shown to increase the rate of first-pass isolation of the left PVs as well as reduce the frequency of pulmonary vein reconnection.^10^ In addition, the ligament of Marshall has been shown to harbor focal triggers of AF,^11^ which may be eliminated with VoMEI. VoMEI also results in local vagal denervation and prevents induction of AF.^12^^,^^13^ Through retrograde infusion of collateral vessels, VoMEI may facilitate roof block and posterior wall isolation.^14^^,^^15^ Achieving durable perimitral block may have direct effects on AF, as well as reducing the risk of postablation perimitral flutter, which was commonly encountered among patients undergoing repeat ablation procedures in the control groups.

### Value of adjuncts beyond PVs

Advances in ablation technologies including catheters capable of very high-power ablation, cryoballoon ablation, and pulsed field ablation have increased the speed, safety, and accessibility of PVI but have not been shown to improve long-term outcomes in patients with persistent AF.^16^, ^17^, ^18^ In this study, patients with nonparoxysmal AF undergoing first-time ablation with PVI had 67% freedom from atrial arrhythmias at 1 year, similar to previously published reports. Consistent with recent randomized controlled trials, the addition of posterior wall isolation in the current study was not associated with improved outcomes.^4^^,^^5^ A prior multicenter randomized study showed that linear lesions at the LA roof and mitral isthmus failed to improve outcomes in patients with persistent AF, as compared with PVI. It is possible that incorporation of strategies such as VoMEI (for more durable protection against perimitral re-entry)^19^ and PWI^20^ vs only a roof line (for prevention of roof-dependent re-entry) might result in improved outcomes.

### Previous studies

Multiple ablation strategies, including the targeting of complex fractionated atrial electrograms, substrate-based ablation, PWI, and empiric linear lesions have failed to demonstrate benefit among patients undergoing ablation of persistent AF.^21^, ^22^, ^23^ Conversely, the Vein of Marshal Ethanol for Untreated Persistent AF (VENUS) trial was able to demonstrate benefit of VoMEI beyond usual ablation approaches using PVI. Several features of this landmark trial limit its interpretation and applicability. For example, performing mitral isthmus ablation was left to the discretion of the operators, resulting in only 85% and 73% of patients in the study and control group, respectively, undergoing mitral isthmus ablation, with fewer patients in these groups achieving mitral block; a follow-up analysis confirmed that attaining mitral block was associated with improved outcomes.^24^ Ablation of non-PVI targets were similarly left to the discretion of the operators, leading to frequent (>90%) ablation of complex fractionated atrial electrograms, and significantly different rates of coronary venous sinus ablation and right atrial ablation/superior vena cava isolation between the control and ablation groups.

The Marshall bundle elimination, Pulmonary vein isolation, and Line completion for ANatomical ablation of persistent atrial fibrillation (Marshall-PLAN) study was a single center study utilizing a fixed ablation approach (VoMEI, CS ablation, PVI, roof, and CTI) and reported a 72% freedom from AF/AT and antiarrhythmic drugs after 1 year, and an 89% success rate after allowing for multiple procedures.^25^ While encouraging, this was a single-arm study without a comparison group. Additionally, this strategy utilized an empiric CTI line, which has not been shown to improve outcomes,^26^ as well as linear ablation at the roof, which may contribute to postablation ATs.^20^ A similar strategy was recently reported by Sang and colleagues.^27^ The true effect size of VoMEI and the optimal ablation lesion set incorporating VoMEI remains unclear, and despite the success of these and other studies, VoMEI remains an underutilized approach, with a recent survey showing that it is incorporated into only 3% of procedures among patients undergoing first-time ablation of persistent AF.^28^

The current study helps address some of these knowledge gaps and further reinforces the utility of VoMEI for persistent AF. To our knowledge, this is the first study to include propensity-matched control groups of the most commonly performed ablation strategies for persistent AF (PVI only and PVI + PWI). The use of a study and control cohorts utilizing an anatomic-based approach with clear endpoints, including demonstration of perimitral block in the study group, reduces complexity and subjectivity compared with previous reports in which extra-PVI ablation was performed at will. The study also suggests limited utility of PWI over PVI only, which has been demonstrated in recent randomized controlled trials.^4^ While the study population was small, a clear and statistically significant benefit was demonstrated regarding AF recurrence rates and the need for fewer procedures. Questions regarding the optimal workflow and assessment of VoMEI efficacy remain,^19^^,^^29^ and future randomized controlled trials are needed to better understand the optimal ablation strategy utilizing VoMEI; nevertheless, these results further clarify the feasibility and effectiveness of VoMEI in improving outcomes of persistent AF ablation.

### Limitations

This was a single center, retrospective study. While the groups were well balanced on key variables through propensity matching, additional confounders are present and randomized controlled trials utilizing standardized ablation approaches are necessary. Duration of AF was unable to be incorporated in the propensity matching due to incomplete database records. The optimal variables for inclusion of propensity matching is not clear but was similar to previously published studies.^30^ Safety, efficacy, and cost-efficiency studies are needed to compare this approach to new technologies such as pulsed field ablation. Ablation strategies did not utilize automated markers of lesion quality in a prespecified fashion, which may have impacted long-term outcomes. Structured approaches to risk factor management were not incorporated into patients’ care. Patients undergoing VoMEI ablation also underwent ablation of inducible tachycardias, as opposed to the anatomy only–based approach performed in the PVI only and PVI + PWI groups. The mean follow-up time varied between groups; however, the survival analysis accounts for these discrepancies and was consistent with improved outcomes in the study group. Randomized controlled trials with prespecified ablation strategies are needed to further understand the safety and efficacy of VoMEI for patients with persistent AF.

## Conclusion

Among patients with persistent AF undergoing first-time ablation procedures, an ablation strategy incorporating VoMEI improved long-term ablation outcomes compared with groups of propensity-matched patients undergoing PVI only or PVI + PWI. Procedural, RF, and ablation times were increased with VoMEI without an increase in procedural complications. Further prospective studies are needed to better understand the optimal ablation approach utilizing VoMEI in the treatment of persistent AF.
